# Clinical comparison of sub-mm high-resolution non-contrast coronary CMR angiography against coronary CT angiography in patients with low-intermediate risk of coronary artery disease: a single center trial

**DOI:** 10.1186/s12968-021-00758-9

**Published:** 2021-05-17

**Authors:** Reza Hajhosseiny, Imran Rashid, Aurélien Bustin, Camila Munoz, Gastao Cruz, Muhummad Sohaib Nazir, Karine Grigoryan, Tevfik F. Ismail, Rebecca Preston, Radhouene Neji, Karl Kunze, Reza Razavi, Amedeo Chiribiri, Pier Giorgio Masci, Ronak Rajani, Claudia Prieto, René M. Botnar

**Affiliations:** 1 School of Biomedical Engineering and Imaging Sciences, King’s College London, 3rdfloor Lambeth Wing, London, SE1 7EH UK; 2 Department of Cardiology, Guy’s and St Thomas’ NHS Foundation Trust, London, UK; 3 Department of Radiology, Guy’s and St Thomas’ NHS Foundation Trust, London, UK; 4 MR Research Collaborations, Siemens Healthcare Limited, Frimley, UK; 5 Escuela de Ingeniería, Pontificia Universidad Católica de Chile, Santiago, Chile

**Keywords:** Coronary artery disease, Coronary magnetic resonance angiography, CMRA, Atherosclerosis, High resolution, Coronary angiography

## Abstract

**Background:**

The widespread clinical application of coronary cardiovascular magnetic resonance (CMR) angiography (CMRA) for the assessment of coronary artery disease (CAD) remains limited due to low scan efficiency leading to prolonged and unpredictable acquisition times; low spatial-resolution; and residual respiratory motion artefacts resulting in limited image quality. To overcome these limitations, we have integrated highly undersampled acquisitions with image-based navigators and non-rigid motion correction to enable high resolution (sub-1 mm^3^) free-breathing, contrast-free 3D whole-heart coronary CMRA with 100% respiratory scan efficiency in a clinically feasible and predictable acquisition time.

**Objectives:**

To evaluate the diagnostic performance of this coronary CMRA framework against coronary computed tomography angiography (CTA) in patients with suspected CAD.

**Methods:**

Consecutive patients (n = 50) with suspected CAD were examined on a 1.5T CMR scanner. We compared the diagnostic accuracy of coronary CMRA against coronary CTA for detecting a ≥ 50% reduction in luminal diameter.

**Results:**

The 50 recruited patients (55 ± 9 years, 33 male) completed coronary CMRA in 10.7 ± 1.4 min. Twelve (24%) had significant CAD on coronary CTA. Coronary CMRA obtained diagnostic image quality in 95% of all, 97% of proximal, 97% of middle and 90% of distal coronary segments. The sensitivity, specificity, positive predictive value, negative predictive value and diagnostic accuracy were: per patient (100%, 74%, 55%, 100% and 80%), per vessel (81%, 88%, 46%, 97% and 88%) and per segment (76%, 95%, 44%, 99% and 94%) respectively.

**Conclusions:**

The high diagnostic image quality and diagnostic performance of coronary CMRA compared against coronary CTA demonstrates the potential of coronary CMRA as a robust and safe non-invasive alternative for excluding significant disease in patients at low-intermediate risk of CAD.

**Supplementary Information:**

The online version contains supplementary material available at 10.1186/s12968-021-00758-9.

## Background

Coronary artery disease (CAD) remains the leading cause of mortality and morbidity worldwide [1]. With a spatial-resolution of ≈ 0.5 mm, negative predictive value of ≈ 99% for excluding significant (≥ 50%) stenosis and low-cost compared to invasive X-ray coronary angiography, coronary computed tomography angiography (CTA) has emerged in clinical guidelines as the non-invasive imaging modality of choice in patients at low-intermediate risk of CAD [2, 3]. Furthermore, fractional flow reserve (FFR_CT_) and plaque characterization (total plaque burden, spotty calcification, plaque attenuation pattern and positive remodeling index) provide additional information [4, 5]. However, in the presence of significant coronary artery calcification, the diagnostic accuracy or coronary CTA is reduced as a result of beam-hardening and calcium-blooming artefacts, while patients with resistant tachycardia/arrhythmias, hypersensitivity to iodinated contrast agents, pregnancy, and inability to follow breath-hold instructions remain challenging to scan [6]. Furthermore, long-term cumulative risk from ionizing radiation, short-term risk of iodinated contrast mediated nephropathy and the lack of ability to readily repeat a study if it is non-diagnostic are further important limitations.

Coronary cardiovascular magnetic resonance angiography (CMRA) may potentially offer a safe, non-invasive, contrast-free and ionizing radiation-free alternative for the anatomical assessment of CAD, which could complement myocardial perfusion, tissue and plaque characterization in a single scanning session [7–9]. Despite the early promise of coronary CMRA [10, 11], clinical application remains limited due to long and unpredictable acquisition times, cumbersome scan planning, low spatial-resolution (≈ 1–2 mm anisotropic) and motion-related image quality degradation.

Recent innovations in cardiovascular magnetic resonance (CMR) technology including respiratory self-gating, trajectory-design, motion-corrected undersampled reconstruction and simplified scan planning have reinvigorated the clinical potential of coronary CMRA [12–17]. Leveraging these breakthroughs, we recently introduced a novel 3D free-breathing, non-contrast, ionizing radiation-free whole-heart coronary CMRA with 100% respiratory scan efficiency at sub-millimeter (≈ 0.9 mm^3^) spatial-resolution in a clinically feasible acquisition time of ≈ 10 min [18]. With this clinical study, we sought to investigate whether this novel coronary CMRA framework could reliably assess for significant CAD in patients at low to intermediate risk against the non-invasive clinical reference standard of coronary CTA.

## Methods

### Study design

This was a prospective single center study where patients undergoing routine coronary CTA at Guy’s and St Thomas’ Hospitals, London, United Kingdom, had a subsequent coronary CMRA scan at the same institution as an research scan. The study was approved by the local institutional review board and the UK National Research Ethics Committee (Reference Number: 230350).

### Study population

**Fig. 1:**
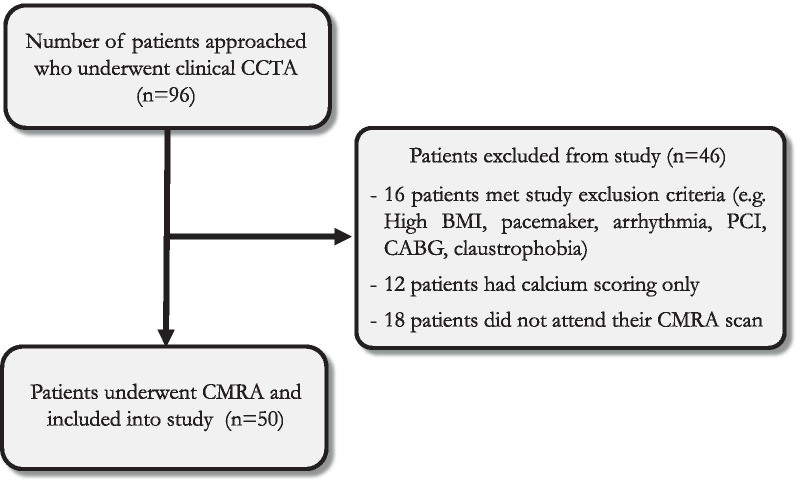
Flowchart of the study subject inclusion. CCTA-Coronary computed tomography angiography; BMI-Body mass index; PCI-Percutaneous coronary intervention; CABG-Coronary artery bypass graft surgery; CMRA-Coronary cardiovascular magnetic resonance angiography

The study population consisted of 50 consecutive patients 35–77 years referred for a clinically indicated coronary CTA between the 1st of October 2018 and 1st of March 2020. Specific exclusion criteria included the following: patients with contraindications for CMR (e.g. pacemaker, cochlear implants, cerebral aneurysm clip, implanted electronic device and claustrophobia), atrial fibrillation, inability to lie flat, body mass index (BMI) > 40 kg/m^2^, previous coronary angioplasty with stent in situ, previous coronary artery bypass grafting or pregnancy. Fig. 1 demonstrates the flow diagram of patient recruitment (Additional file 1).

### Study protocol

All patients were recruited from the outpatient coronary CTA lists. All coronary CMRA scans were performed within a median of 11 days of the preceding coronary CTA scan. In the absence of contraindications, each patient was treated with intravenous metoprolol (Betaloc ®, AstraZeneca, Cambridge, United Kingdom), in 5 mg increments via a peripheral intravenous cannula with a maximum dose of 30 mg aiming for a target heart rate (HR) < 65 bpm in order to maximize the diastolic acquisition window, reduce HR variability and cardiac motion artefacts. All patients were given 800 mcg of sublingual glyceryl trinitrate to promote coronary vasodilation unless the systolic blood pressure was less than 100 mmHg.

### Coronary cardiovascular magnetic resonance angiography

**Fig. 2:**
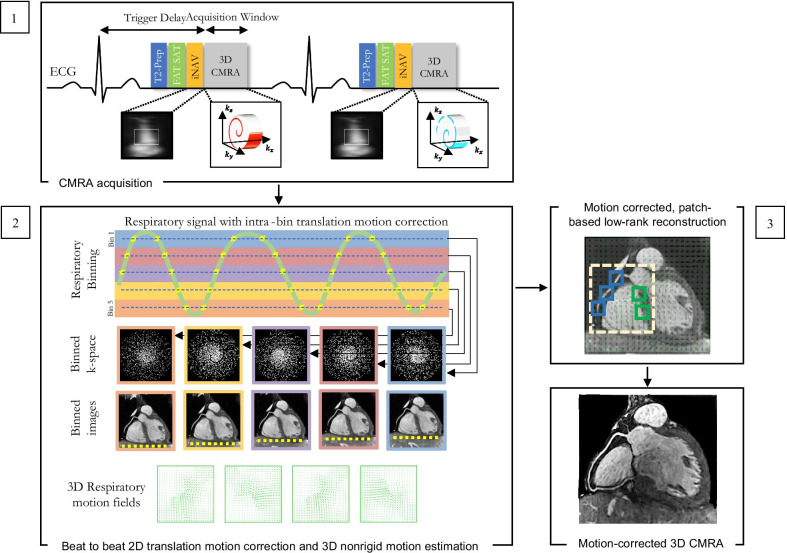
Schematic overview of the proposed accelerated free-breathing 3D coronary CMRA acquisition with sub-millimeter isotropic resolution. **1** Coronary CMRA acquisition is performed with an undersampled 3D variable density spiral-like Cartesian trajectory with golden angle between spiral-like interleaves (VD-CASPR), preceded by 2D image navigators (iNAV) to allow for 100% scan efficiency and beat-to-beat translational respiratory-induced motion correction of the heart. **2** Foot-head respiratory signal is estimated from the 2D iNAVs and used to assign the acquired data into 5 respiratory bins and translation-corrected respiratory bins. Subsequent reconstruction of each bin is performed using soft-gated iterative SENSE and 3D non-rigid motion fields are then estimated from the 5 reconstructed datasets. **3** The final 3D whole-heart motion-corrected CMRA image is obtained using PROST non-rigid motion-compensated reconstruction. CMRA-Coronary cardiovascular magnetic resonance angiography; PROST-Patch-based undersampled reconstruction

All coronary CMRA acquisitions were performed on a 1.5T CMR scanner (MAGNETOM Aera, Siemens Healthineers, Erlangen, Germany) with a dedicated 32-channel spine coil and an 18-channel body coil. A 3-lead vector electrocardiogram (ECG) was used for cardiac synchronization. A multislice survey using balance steady state free precession (bSSFP) acquisition was performed in the axial, coronal and sagittal planes. To determine the mid-diastolic rest period, a free-breathing axial cine was acquired in a pseudo 4-chamber orientation. The cine images were used to determine the optimal patient-specific trigger delay and image acquisition window during the mid-diastolic rest period that corresponds with minimized motion of the visualized right coronary artery. Thereafter, an undersampled free-breathing 3D whole-heart ECG-triggered, bSSFP sequence with a 3D variable density spiral-like Cartesian trajectory with golden-angle rotation was employed [18]. A low-resolution 2D image navigator (iNAV) preceded each spiral-like interleave to allow for 100% respiratory scan efficiency, predictable scan time and 2D translational motion correction of the heart on a beat-to-beat basis. The 2D iNAV images were obtained by spatially encoding the startup profiles of the bSSFP sequence [19]. A spectrally selective Spectral Presaturation with Inversion Recovery (SPIR) fat saturation pulse with a constant flip angle of 130° was used to improve coronary depiction and minimize fat-related aliasing artefacts. An adiabatic T2 preparation pulse (duration = 40 ms) [20, 21] was played out at each heartbeat in order to enhance the contrast between blood and cardiac muscle and avoid the use of extracellular contrast agents. The image reconstruction framework consists of 3 different steps (i) beat-to-beat respiratory binning and intra-bin translational motion correction using iNAV; (ii) bin-to-bin 3D non-rigid motion estimation; and (iii) non-rigid motion-corrected 3D patch-based low-rank reconstruction (PROST) [18]. A schematic overview of the coronary CMRA framework is shown in Fig. 2 and has been described in detail in Bustin et al. [18].

### Multidetector coronary computed tomography angiography

All coronary CTA studies were performed using a third generation dual-source CT (Siemens Healthineers) with ECG synchronization. A combined automated tube potential selection software (CAREkV, Siemens Healthineers) and current selection algorithm (CAREDose 4D, Siemens Healthineers) was utilized with a reference peak tube voltage of 120 kV and reference mA of 280. Intravenous metoprolol in 5 mg incremental doses was used where required to achieve a HR < 65 bpm and 800 mcg sublingual gyleryl trinitrate was administered to all patients. All scans were acquired by prospectively ECG-triggered axial acquisition scan mode extending from the carina to the inferior aspect of the heart upon an inspiratory breath hold with a grantry rotation time of 250 ms. The ECG pulsing and padding range was automatically determined by the scanner with arrhythmia rejection enabled (Adaptive Cardio Sequential). In all cases Omnique 370 mg/ml was injected at a rate of 5–6 mls/second followed by a 75 mls mixed bolus of contrast: saline (35%:65%) and a 25 mls saline chaser. Bolus tracking was used with the scans triggered once a minimum of 110 Hounsfield Units was achieved in the proximal descending aorta. All images were reconstructed using ADMIRE strength 2 (Advanced Modeled Iterative Reconstruction, Siemens Healthineers) using a medium-smooth dedicated heart kernel (Bv40 d).

### Coronary CMRA and coronary CTA Data analysis

Both coronary CMRA and coronary CTA 3D datasets were analyzed using a 9 coronary segment model, described in previous coronary CMRA studies [10, 15, 16]. Briefly, this divides the coronary artery tree into the following segments: left main stem (LM) coronary artery, left anterior descending (LAD) coronary artery (proximal, middle and distal), right coronary artery (RCA) (proximal, middle and distal) and left circumflex (LCx) artery (proximal and distal). To assess diagnostic performance, significant coronary stenosis was visually defined as luminal narrowing of ≥ 50% in each of the 9 segments using an intention-to-read approach. The image quality of coronary CTA and coronary CMRA images (3D whole-heart dataset and individual vessels) was evaluated using the following scale: 0, non-diagnostic; 1, poor (limited coronary vessel visibility or noisy image); 2, average (coronary vessel visible but diagnostic confidence low); 3, good (coronary artery adequately visualized and diagnostic quality image); and 4, excellent (coronary artery clearly depicted). All coronary segments were included for evaluation regardless of the image quality to avoid overestimation of the diagnostic accuracy.

All coronary CTA scans were indepedently analysed by a Society of Cardiovascular Computed Tomography (SCCT) and European Association of Cardiovascular Imaging (EACVI) level III verified practitioner with more than 10 years of experience in clinical cardiovascular CT (RR) who was blinded to the patient clinical information and coronary CMRA findings. All coronary CTA data analysis was performed on an Aquarius iNtuition Edition workstation version 4.4.13.P3 (Terarecon, Durham, North Carolina, USA). The coronary CMRA scans were analysed by two independent experts with Society for Cardiovascular Magnetic Resonance (SCMR) and EACVI level III accreditation and more than 14 years (PGM) and 5 years (MSN) of experience in clinical CMR who were blinded to the patient clinical information and coronary CTA findings. Inter-observer agreement of the visual scores was performed using Cohen’s kappa coefficient where a coefficient less than 0.4 was considered poor, between 0.4 and 0.75 good, and higher than 0.75 excellent agreement. In cases in which there was a disagreement between the observers, a consensus approach was used. All coronary CMRA data analysis was performed on Osirix software (Osirix, Pixmeo SARL, Bernex, Switzerland).

In order to evaluate the impact of HR on coronary CMRA image quality, the patients were divided into 2 groups based on HR as follows: group 1, HR < 70 beats/min and group 2, HR ≥ 70 beats/min. We also analysed the effect of coronary CMRA acquisition time on image quality by dividing patients into 2 groups based on acquisition time as follows: group A, acquisition time < 10 min and group B, acquisition time ≥ 10 min.

### Statistical analysis

All statistical analysis was performed using GraphPad Prism 8 (version 8.4.1, GraphPad Software, San Diego, California, USA). For each individual patient, coronary vessel and coronary segment, the sensitivity, specificity, positive predictive value (PPV), negative predictive value (NPV), and diagnostic accuracy was calculated with a 95% confidence interval (CI) for coronary CMRA as compared with coronary CTA as the non-invasive clinical reference standard. Continuous variables are presented as mean ± standard deviation and assessed using a two-tailed t-test. Ordinal categorical variables are presented as median and interquartile range and assessed using either a Wilcoxon signed rank test or Mann-Whitney U test. Nominal categorical variables in comparison of cross tables were assessed using Fisher’s exact test or the chi-square test. A two-tailed p value ≤ 0.05 was considered statistically significant.

## Results

In total 50 patients underwent both coronary CTA and coronary CMRA. Coronary CMRA acquisitions were completed in 10.7 ± 1.4 mins (range 8.0–13.3 mins), with 100% respiratory scan efficiency. All coronary CMRA acquisitions were performed in diastole with an average acquisition window of 88 ± 8 ms (range 81–111 ms). The average HR for patients undergoing a coronary CTA scan was 58 ± 9 beats/min compared with 64 ± 7 beats/min for patients undergoing a coronary CMRA scan ( p< 0.001). The average dose of intravenous metoprolol prior to coronary CTA was 9 ± 11 mg compared with 11 ± 6 mg prior to coronary CMRA (p = 0.497). There was excellent agreement between the CMR observers for the stenosis and image quality scoring, with a kappa coefficient of 0.75 and 0.79 respectively. Table 1 summarizes the baseline patient characteristics.

**Table 1:** Baseline patient characteristics

Characteristics	All patients (n = 50)
Male/Female n (%)	33/17 (66/34)
Age (years) ± SD	55.2 ± 9.4
Range (years)	35–77
Height (cm) ± SD	171.3 ± 10.3
Range (cm)	150–189
Weight (kg) ± SD	83.0 ± 14.5
Range (kg)	52–112
BMI (kg/m^2^) ± SD	28.3 ± 4.4
Range (kg/m^2^)	19–38
Diabetes, n (%)	9 (18)
Hypertension, n (%)	14 (28)
Smoker, n (%)	8 (16)
Hyperlipidemia, n (%)	12 (24)
Family history of CAD, n (%)	16 (32)
Indication for scan	
Chest Pain, n (%)	42 (84)
Shortness of breath, n (%)	4 (8)
Abnormal ECG, n (%)	3 (6)
Heart Failure/Cardiomyopathy, n (%)	1 (2)
Significant disease prevalence, n (%)	12 (24)
3 vessel disease, n (%)	1 (2)
2 vessel disease, n (%)	3 (6)
1 vessel disease, n (%)	8 (16)
CAD-RADS score	
0–2 (< 50% stenosis), n (%)	38 (76%)
3 (50–69% stenosis), n (%)	4 (8%)
4 (70–99% stenosis), n (%)	7 (14%)
5 (Occluded), n (%)	1 (2%)
CAC score prevalence	
0, n (%)	25 (50)
1–100, n (%)	16 (32)
101–300, n (%)	5 (10)
> 300, n (%)	4 (8)

Data are expressed as mean ± SD or n (%). BMI, Body mass index; ECG, Electrocardiogram; CAC, Coronary artery calcium; CAD-RADS, Coronary Artery Disease-Reporting and Data System

### Image quality

**Fig. 3:**
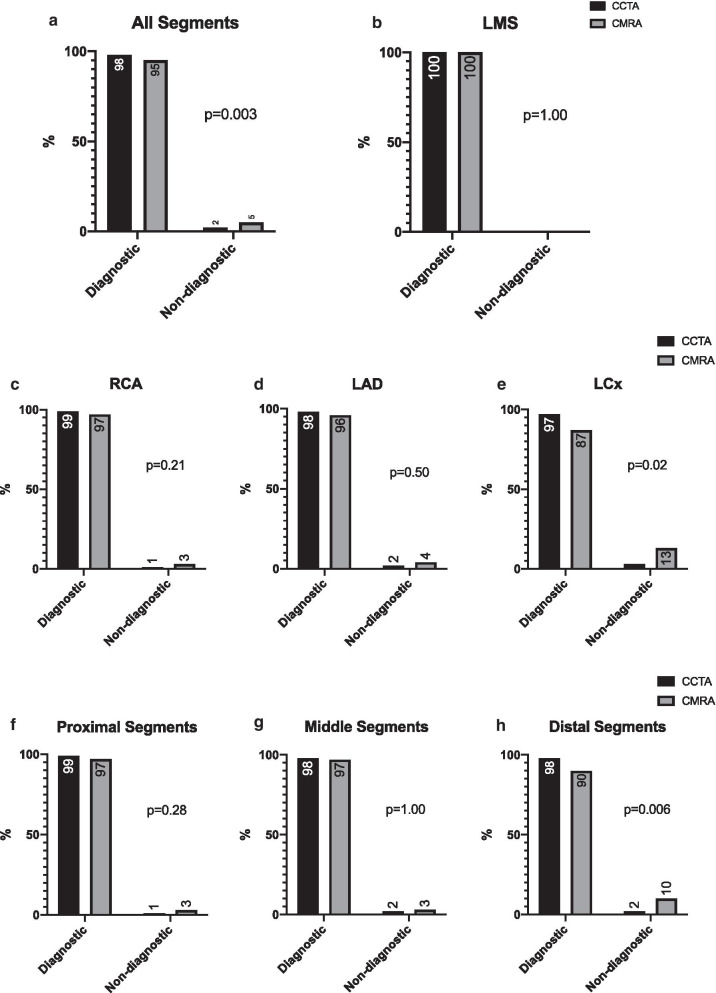
Percentage of all (**a**), left main (LM) coronary artery (**b**), right coronary artery (RCA) (**c**), left anterior descending (LAD) coronary artery (**d**), left circumflex (LCx) coronary artery (**e**), proximal (**f**), middle (**g**) and distal (**h**) coronary segments that were of diagnostic quality for coronary CTA and coronary CMRA datasets

**Fig. 4:**
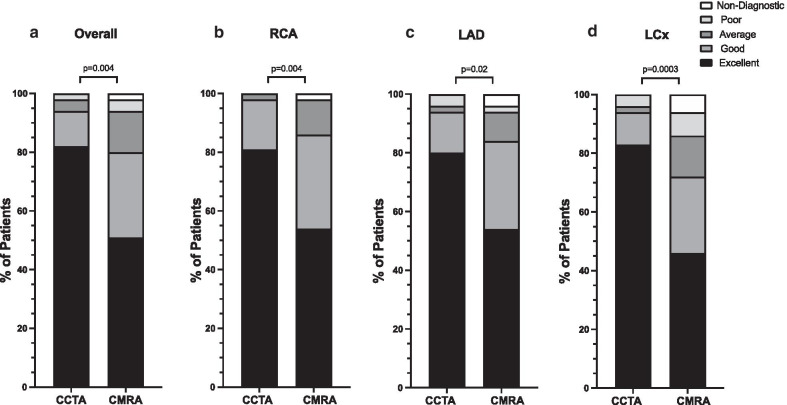
Distribution of image quality scores for coronary CTA vs. coronary CMRA. **a** The overall 3D whole-heart dataset, **b** RCA, **c** LAD and **d** LCx

In total, 443/450 (98%) of coronary CTA segments were deemed diagnostic compared with 426/450 (95%) of coronary CMRA segments (p = 0.003) (Fig. 3a), while all LM segments were diagnostic for both coronary CTA and coronary CMRA datasets (p = 1.00) (Fig. 3b). Overall, 99%, 98% and 97% of RCA, LAD and LCx coronary CTA segments were of diagnostic quality compared with 97%, 96% and 87% of coronary CMRA segments respectively (Fig. 3c–e). Furthermore, 99%, 98% and 98% of proximal, middle and distal coronary CTA segments were of diagnostic quality compared with 97%, 97% and 90% of coronary CMRA segments respectively (Fig. 3f–h). Of all non-diagnostic coronary CMRA segments, 13/24 (54%) were located in the LCx and 15/24 (63%) were distal segments. Fig. 4 summarizes the image quality scores for the overall 3D dataset as well as the three coronary vessels.

### Impact of heart rate and acquisition time on image quality scores

There were 38 patients with a HR < 70 beats/min (mean HR 61 ± 6 beats/min, range 50 to 69 beats/min) and 12 patients with a HR ≥ 70 beats/min (mean HR 72 ± 2 beats/min, range 70 to 75 beats/min). There were 15 patients with an acquisition time < 10 min (mean acquisition time 9.2 ± 0.5 min, range 8.0 to 9.9 min) and 35 patients with an acquisition time ≥ 10 min (mean acquisition time 11.5 ± 1.1 min, range 10.1 to 13.3 min).

**Fig. 5:**
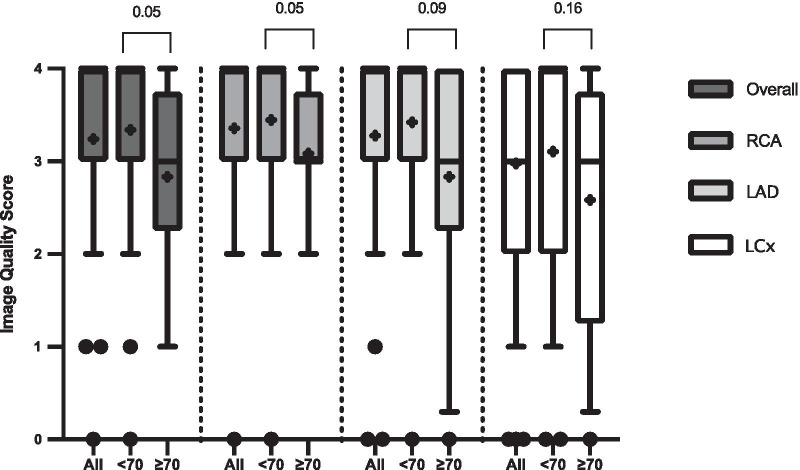
Impact of heart rate on image quality score for the coronary CMRA scans. Box and whisker plots are expressed as median, interquartile range, 10th and 90th percentile of values. The “+” sign within the box plots represents the mean image quality value of each dataset. “All” represents all patients, “< 70” represents patients with a heart rate of < 70 beats/min and “≥ 70 represents patients with a heart rate of ≥ 70 beats/min. Overall-Overall 3D whole-heart dataset

**Fig. 6:**
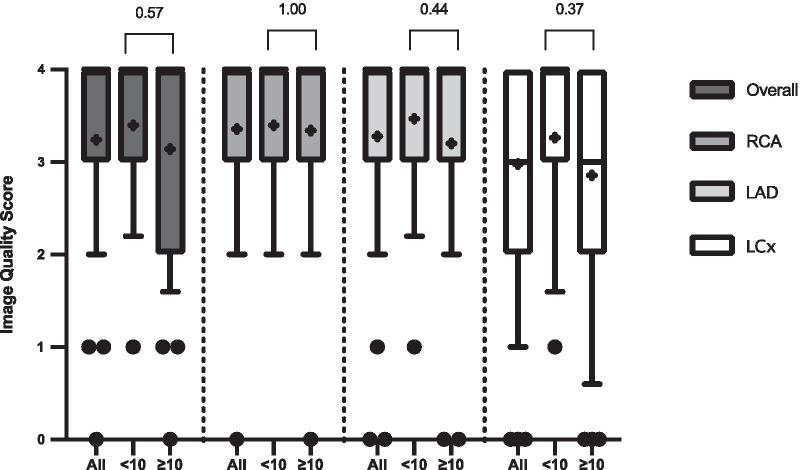
Impact of scan acquisition time on image quality score for the coronary CMRA scans. Box and whisker plots are expressed as median, interquartile range, 10th and 90th percentile of values. The “+” sign within the box blots represents the mean image quality value of each dataset. “All” represents all patients, “< 10” represents patients with an acquisition time of < 10 min and “≥  10 represents patients with an an acquisition time of ≥ 10 min. Overall-Overall 3D whole-heart dataset

Figures 5 and 6 summarize the impact of HR and acquisition time on coronary CMRA image quality scores for the 3D whole-heart dataset and individual vessels respectively. Patients with a HR of < 70 beats/min had significantly higher image quality scores for both the overall 3D whole-heart dataset and the RCA compared with patients with a HR of ≥ 70 beats/min (p = 0.05). While there was a trend of improved image quality for the LAD and LCx arteries for HR < 70 beats/min, this did not reach significance (p = 0.09 and p = 0.16 respectively). Similarly, there was a non-significant trend of improved image quality for patients with a coronary CMRA acquisition time of < 10 min compared with patients with a coronary CMRA acquisition time of ≥ 10 min.

### Agreement between coronary MRA and coronary CTA

The clinical performance of coronary CMRA compared with coronary CTA is summarized in Table 2. All vessels and segments of diagnostic quality were included in the analysis. The sensitivity, specificity, PPV, NPV and diagnostic accuracy of coronary CMRA for detecting significant CAD were as follows: per patient 100% (95% CI 76–100%), 74% (95% CI 58–85%), 55% (95% CI 35–73%), 100% (95% CI 88–100%) and 80% (95% CI 67–89%) respectively; per vessel 81% (95% CI 57–93%), 88% (95% CI 82–93%), 46% (95% CI 30–64%), 97% (95% CI 93–99%) and 88% (95% CI 81–92%) respectively; per segment 76% (95% CI 55–89%), 95% (95% CI 92–97%), 44% (95% CI 30–60%), 99% (95% CI 97–99%) and 94% (95% CI 91–96%) respectively.

**Table 2:** Diagnostic performance of 3D whole-heart coronary CMRA compared with coronary CTA

	Sensitivity	Specificity	PPV	NPV	Accuracy
**Per Patient**	**100 (12/12) [76–100]**	**74 (28/38) [58–85]**	**55 (12/22) [35–73]**	**100 (28/28) [88–100]**	**80 (40/50) [67–89]**
**Per Vesse**l	**81 (13/16) [57–93]**	**88 (115/130) [82–93]**	**46 (13/28) [30–64]**	**97 (115/118) [93–99]**	**88 (128/146) [81–92]**
RCA	60 (3/5) [23–93]	91 (41/45) [79–96]	43 (3/7) [16–75]	95 (41/43) [85–99]	88 (44/50) [76–94]
LAD	88 (7/8) [53–99]	86 (36/42) [72–93]	54 (7/13) [29–77]	97 (36/37) [86–100]	86 (43/50) [74–93]
LCx	100 (3/3) [44–100]	91 (39/43) [78–96]	43 (3/7) [16–75]	100 (39/39) [91–100]	91 (42/46) [80–97]
**LM**	**N/A (0/0)**	**98 (49/50) [90–100]**	**0 (0/1) [0–95]**	**100 (49/49) [93–100]**	**98 (49/50) [90–100]**
**Per Segment**	**76 (16/21) [55–89]**	**95 (378/398) [92–97]**	**44 (16/36) [30–60]**	**99 (378/383) [97–99]**	**94 (394/419) [91–96]**
Proximal	70 (7/10) [40–89]	95 (173/182) [91–97]	44 (7/16) [23–67]	98 (173/176) [95–99]	94 (180/192) [89–96]
Middle	100 (6/6) [61–100]	92 (82/89) [85–96]	46 (6/13) [23–71]	100 (82/82) [95–100]	93 (88/95) [86–96]
Distal	60 (3/5) [23–93]	97 (123/127) [92–99]	43 (3/7) [16–75]	98 (123/125) [94–100]	95 (126/132) [90–98]

Significant values are indicated in bold

% (raw data) [95% confidence interval]

RCA, Right coronary artery; LAD, Left anterior descdending coronary artery; LCx, Left circumflex coronary artery; LM, Left main coronary artery; PPV, Positive predictive value; NPV, Negative predictive value

**Fig. 7:**
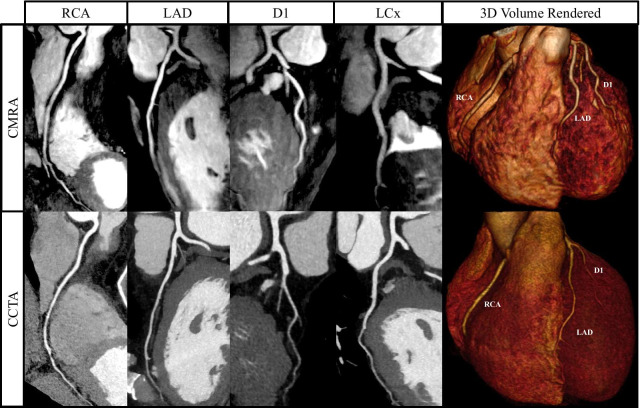
Curved multiplanar reformat and 3D volume rendered non-contrast coronary CMRA and contrast enhanced coronary CTA in a 54 year old male with no significant stenosis. D1-First diagonal coronary artery

**Fig. 8:**
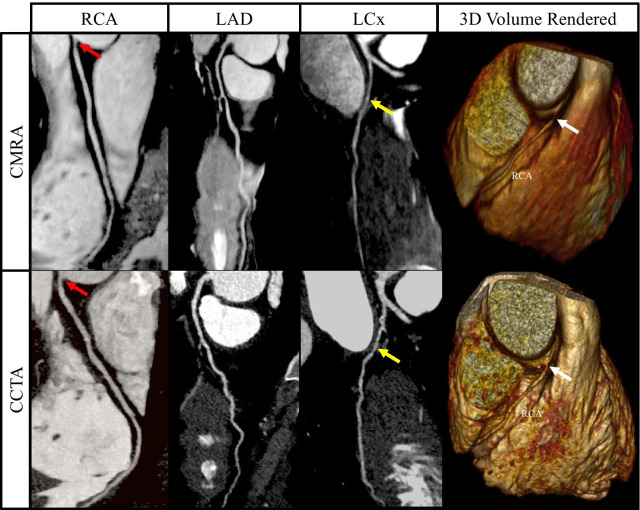
Curved multiplanar reformat and 3D volume rendered non-contrast coronary CMRA and contrast enhanced coronary CTA in a 44 year old male with > 50% non-calcified stenosis in the ostial RCA (red arrows). This can also be seen in the 3D volume rendered images (white arrows). The yellow arrows represent a > 50% stenosis in the proximal/mid LCx

**Fig. 9:**
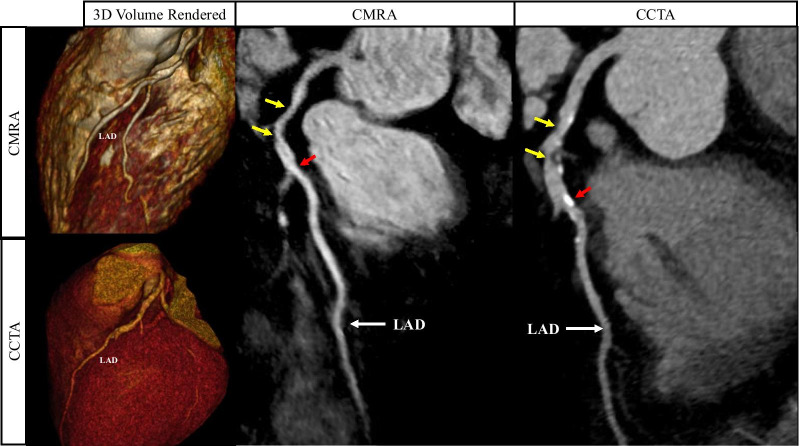
Curved multiplanar reformat and 3D volume rendered non-contrast coronary CMRA and contrast enhanced coronary CTA in a 60 year old male with > 50% partially calcified stenosis in the proximal to mid LAD either side of the first diagonal artery (yellow arrows). The red arrows represent a focal calcified < 50% stenosis just distal to the second diagonal artery

**Fig. 10:**
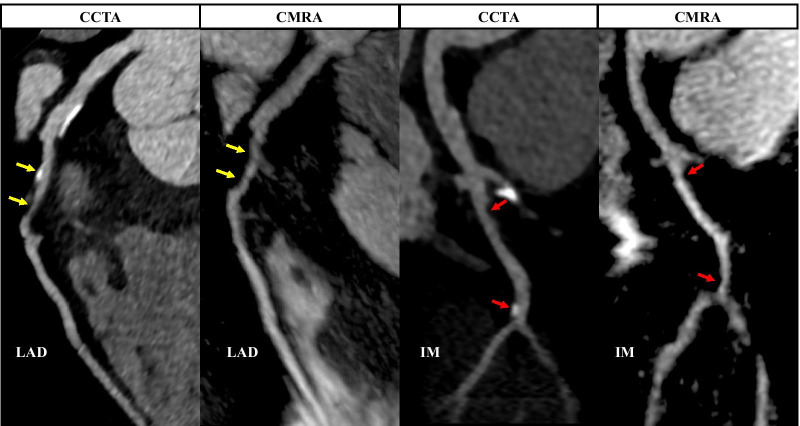
Curved multiplanar reformat non-contrast coronary CMRA and contrast enhanced coronary CTA in a 57 year old male with > 50% partially calcified stenosis in the proximal LAD (yellow arrows). The red arrows represent focal < 50% stenosis in the proximal and distal ramus intermedius artery. IM-Ramus intermedius artery

Example images from selected patients with suspected CAD, with both coronary CMRA and coronary CTA are shown in Figures 7, 8, 9, 10 and Additional file 2: Video 1.

## Discussion

In this prospective, single center trial of 50 consecutive patients with suspected CAD, we assessed for the first time, the diagnostic ability of our recently proposed high-resolution, non-contrast coronary CMRA framework [18] in excluding significant CAD compared against the non-invasive clinical anatomical reference standard of coronary CTA. We applied an intention-to-read analysis approach by including all diagnostic coronary segments regardless of image quality.

We achieved a predictable and clinically feasible average acquisition time of approximately 10 mins, which is comparable to previously published clinical studies despite a significantly higher spatial-resolution (0.9 mm^3^). Whilst including all coronary segments for analysis, only 24/450 (5%) of all coronary CMRA segments were non-diagnostic, with an overall median image quality score of 4.0 (IQR 3.0–4.0), representing “good to excellent” on the image quality scale.

The sensitivity of our coronary CMRA framework was comparable to previous coronary CMRA studies which had a range of 71–94% per patient, 65–93% per vessel and 46–92% per segment respectively [10, 11, 14–16, 22, 23]. Furthermore, our coronary CMRA approach demonstrated a high specificity and NPV per patient (74% and 100%), per vessel (88% and 97%) and per segment (95% and 99%). In addition, we demonstrated a very high specificity and NPV for excluding LM (98% and 100%), proximal segment (95% and 98%), middle segment (92% and 100%) and distal segment stenoses (97% and 98%), indicating the clinical potential of our proposed coronary CMRA framework in patients with low-intermediate risk of CAD.

To our knowledge, this is the first clinical study to assess the diagnostic performance of a 3D contrast-free coronary CMRA approach using similar patient preparation as coronary CTA that enables a predictable scan time of approximately 10 min for 0.9 mm^3^ spatial-resolution. This was achieved by employing a robust motion corrected free-breathing acquisition with 100% respiratory scan efficiency, using image based navigation for 2D translational motion estimation and respiratory data binning combined with 3D non-rigid motion compensated reconstruction based on a 3-4 fold undersampled Cartesian acquisition and a patched based low rank reconstruction. Yang et al [22] and more recently Sun et al. [23] utilized a conventional diaphragmatic respiratory navigator in conjunction with a slow infusion of gadobenate dimeglumine to obtain 3D whole-heart coronary CMRA images. Sacrificing spatial-resolution (1.3 mm^3^ and 1.3 × 1.3 × 1.8 mm respectively), they achieved an average acquisition time of 9.0-9.5 min (acquisition window of 70–135). However, average respiratory scan efficiency was 35–36%, 9–10% of patients were unable to complete their scan to due to unpredictable scan times and 12–14% of all segments were excluded prior to analysis. Similarly, Kato et al. [11] combined the same protocol in a non-contrast study (spatial-resolution 1.3 × 1.3 × 1.7 mm), achieving an average acquisition time of 9.5 min (acquisition window of 131 ± 40 ms). However, the respiratory scan efficiency was 37%, 8% of patients were unable to complete the scan, due to unpredictable scan times and 10% of all segments were excluded prior to analysis. To overcome the unpredictability of the conventional diaphragmatic navigator, Piccini et al. [15] acquired self-navigated 3D whole-heart coronary CMRA with 100% respiratory scan efficiency at 1.15 mm^3^ spatial-resolution in a predictable acquisition time of 7.4 min ± 1.9 min. However, this was a contrast-enhanced study and 8% of proximal segments, 16% of middle segments and 44% of distal segments could not be visualized, severely hampering the diagnostic abilities of this framework. He et al. [14] improved on this by using higher field strength (3T) and spatial-resolution (1 mm^3^), achieving a predictable acquisition time of 7.8 ± 0.8 min. However, this was a contrast-enhanced study and they excluded 12% of all segments from analysis due to limited diagnostic quality. Another disadvantage of self-navigation is the difficulty in separating moving (e.g. heart) from static (e.g. chest wall) tissue, which may reduce the motion correction performance [19]. iNAVs overcome some of the limitations of respiratory self-navigation, e.g. separating moving tissue from static tissue during motion estimation to enable beat-to-beat translational motion correction with or without respiratory gating. We have previously investigated the clinical robustness of the iNAV based coronary CMRA for assessing CAD [16]. However, this framework utilized contrast-enhancement and fixed 50% respiratory gating. The spatial-resolution was 1.3 mm^3^ and the average acquisition time was 7.3 ± 0.5 min (acquisition window of 118 ± 38 ms). However, 9% of all coronary segments were excluded from the analysis due to low spatial-resolution. In contrast, 100% of patients in the present study completed image acquisition and no coronary segments were excluded prior to analysis, despite the significantly higher spatial-resolution. Unique to this framework is the combination of the 3D non-rigid motion correction framework with the golden-step variable density spiral-like Cartesian trajectory which enables further improvement in motion correction and image quality at 100% scan efficiency, particularly at high spatial-resolution. Furthermore, the 3D patch-based reconstruction technique enables sub-1mm spatial-resolution which was not clinically feasible previously.

### Coronary CMRA vs coronary CTA

In a small pilot study of 32 selected patients with calcified plaque on coronary CTA with corresponding invasive X-ray coronary angiography, Liu et al. [24] performed non-contrast 3D vessel targeted and 3D whole-heart coronary CMRA on a 1.5T scanner using conventional diaphragmatic navigator respiratory gating. The specificity for significant stenosis on coronary CMRA was significantly higher than coronary CTA compared against the gold-standard of invasive coronary angiography (75% vs 48%, respectively; p = 0.002). In a larger study of 120 consecutive patients with known or suspected CAD, Hamdan et al. [25] compared the diagnostic performance of conventional diaphragmatic navigated contrast-enhanced coronary CMRA on a 3T scanner against coronary CTA in patients who also underwent invasive coronary angiography. The diagnostic performance of coronary CMRA and coronary CTA was similar on a per patient and per vessel basis, with a sensitivity of 87% and 90% respectively and specificity of 77% and 83% respectively. It is worth noting that both coronary CT and coronary CMR technology have leaped significantly since these studies were published.

### Study limitations

We observed a significantly lower average HR in patients who underwent a coronary CTA compared to the same patients who subsequently underwent a coronary CMRA scan. Furthermore, only 4% of patients on the day of their coronary CTA scan had a HR of ≥  70 beats/mins compared with 24% of patients on the day of their coronary CMRA scan (p = 0.008). We also observed a significantly higher coronary CMRA image quality score for the overall 3D whole-heart dataset and RCA in patients with a HR < 70 beats/mins compared with patients with a HR of ≥  70 beats/mins, although the numbers are limited. This discrepancy could in part be explained by the fact that patients who attended their clinical coronary CTA scan were pre-conditioned with three days of oral metoprolol leading up to the date of their scan. A low HR directly correlates with a longer diastolic resting period of the heart and lower likelihood of cardiac motion artefacts during the coronary CMRA acquisition [26, 27]. Furthermore, metoprolol suppresses both arrhythmia and ectopic beats [28], which is crucial for free-breathing 3D whole-heart coronary CMRA acquisition whereby data is acquired over several consecutive heart beats. Additionally, beta blockers have been shown to significantly reduce HR variability in patients undergoing coronary CTA, with significant improvements in image quality and reduction of artefacts [29].

This study compared the diagnostic performance of coronary CMRA against coronary CTA, without comparison against invasive X-ray coronary angiography. Whilst coronary CTA has an excellent NPV for excluding significant CAD, its specificity is still limited as described in the studies by Liu et al. [24] and Hamdan et al. [25]. Furthermore, in our center, patients with very high coronary artery calcium scores (as assessed by the clinical team on the day of the scan) were referred directly for an invasive X-ray coronary angiogram, without performing a contrast enhanced coronary CTA, in order to avoid unnecessary exposure of patients to ionising radiation and iodine contrast agents. Furthermore, the PPV of this coronary CMRA framework was comparatively low compared with previously published studies. This could potentially be explained by the relatively small number of patients in this study, compounded by the low prevalence of severe stenosis in this patient cohort, possible overcautious analysis as well as the absence of invasive X-ray coronary angiography as the arbiter. Therefore, larger multi-center studies should assess the performance of this novel coronary CMRA framework against both coronary CTA and invasive X-ray coronary angiography.

This coronary CMRA framework is currently not applicable to patients who met our exclusion criteria. Future studies should aim to include as many of these patients as possible in order to capture the full spectrum of patients with suspected CAD, e.g. through arrhythmia rejection reconstruction and systolic imaging in atrial fibrillation.

### Future developments

Despite the promising results of this coronary CMRA framework, there is nevertheless room for further technical developments and improvement of image quality. Future iterations of this framework could potentially incorporate automatic detection of the iNAV position and trigger delay in order to simplify the planning process, minimise user input and reduce errors. The 2D iNAV could also be extended to enable 3D translational motion estimation, without requiring additional imaging or increasing scan time. Furthermore, deep learning neural networks could be employed to enable undersampled and/or super resolution reconstruction, thereby significantly accelerating image acquisition and reconstruction, whilst also allowing motion correction directly from the acquired k-space data, which is less sensitive to undersampling artefacts and may allow even higher undersampling factors. Recent proof-of-concept studies [30–32] demonstrate the feasibility of obtaining sub-millimeter resolution CMRA in less than 1 min or even within a breathhold thus approaching the acquisition time of coronary CTA.

## Conclusions

In this clinical study, we have clinically validated a highly novel and robust coronary CMRA framework, which synergistically combines major advances in motion estimation, k-space sampling and motion corrected undersampled reconstruction, and thus enables isotropic spatial resolution within a predictable and clinically feasible scan time of approximately 10 min. It is contrast-free, does not use ionizing radiation and was well tolerated by all patients, achieving high diagnostic accuracy for excluding significant disease in patients at low-intermediate risk of CAD.

## Supplementary Information

**Additional file 1. **Overview of the proposed accelerated free-breathing 3D coronary cardiovascular magnetic resonance angiography (CMRA) acquisition with sub-millimeter spatial-resolution, 100% scan efficiency and predictable acquisition time.

**Additional file 2. **Movie through the short axis of non-contrast coronary CMRA (right column) and contrast enhanced coronary CTA (left column) in three patients presenting with chest pain.

## Data Availability

Not applicable.

## Abbreviations

BMI: Body mass index
bSSFP: Balanced steady state free precession
CABG: Coronary artery bypass graft surgery
CAD: Coronary artery disease
CMR: Cardiovascular magnetic resonance
CMRA: Cardiovascular magnetic resonance angiography
CTA: Computed tomography angiography
EACVI: European Association of Cardiovascular Imaging
ECG: Electrocardiogram
FFR_CT_: Fractional flow reserve by computed tomography
HR: Heart rate
iNAV: Image navigator
LAD: Left anterior descending coronary artery
LCx: Left circumflex coronary artery
LM: Left main coronary artery
NPV: Negative predictive value
PPV: Positive predictive value
RCA: Right coronary artery
SCCT: Society of Cardiac Computed Tomography
SCMR: Society for Cardiovascular Magnetic Resonance
SPIR: Spectral presaturation with inversion recovery
