# Supplementary material for: Clinical comparison of sub-mm high-resolution non-contrast coronary CMR angiography against coronary CT angiography in patients with low-intermediate risk of coronary artery disease: a single center trial
Source: J Cardiovasc Magn Reson. 2021 May 17;23:57. doi: 10.1186/s12968-021-00758-9 (PMC8127202; doi:10.1186/s12968-021-00758-9)
Supplement: Supplementary file 1 — Additional file 1. Overview of the proposed accelerated free-breathing 3D coronary cardiovascular magnetic resonance angiography (CMRA) acquisition with sub-millimeter spatial-resolution, 100% scan efficiency and predictable acquisition time. [file 12968_2021_758_MOESM1_ESM.pptx]

## Slide 1
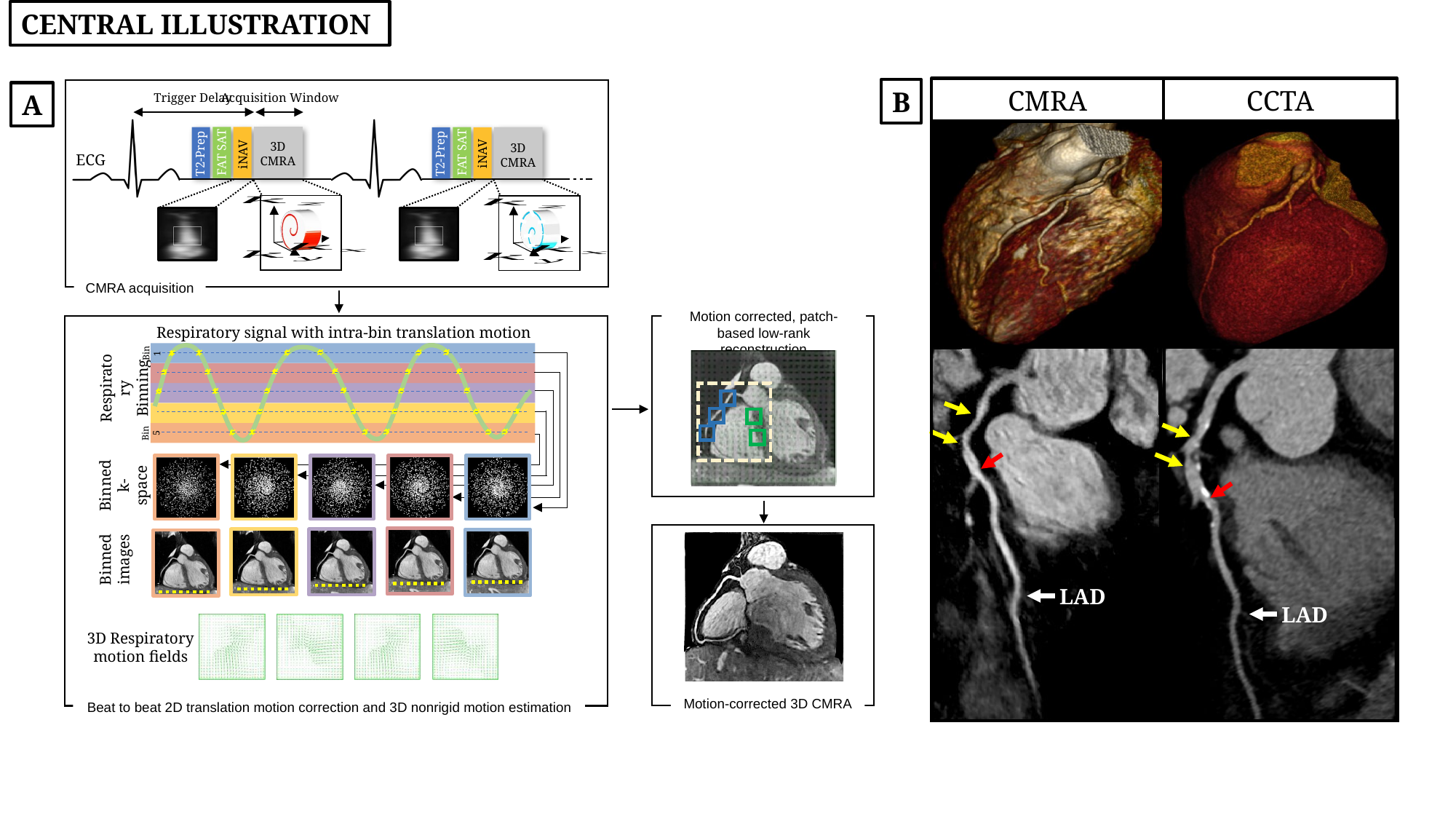

CENTRAL ILLUSTRATION
CMRA
CCTA
B
A
Acquisition Window
Trigger Delay
3D
CMRA
3D
CMRA
FAT SAT
FAT SAT
iNAV
T2-Prep
T2-Prep
iNAV
ECG
CMRA acquisition
Motion corrected, patch-based low-rank reconstruction
Respiratory signal with intra-bin translation motion correction
Bin 1
Respiratory Binning
Bin 5
Binned k-space
Binned images
LAD
LAD
3D Respiratory motion fields
Motion-corrected 3D CMRA
Beat to beat 2D translation motion correction and 3D nonrigid motion estimation
